# General Awareness Regarding Dual-Energy X-ray Absorptiometry (DEXA) Scan and Osteoporosis in Saudi Arabia: A Cross-Sectional Study

**DOI:** 10.7759/cureus.83165

**Published:** 2025-04-29

**Authors:** Ziyad A Almushayti, Anwar H Alhatlani, Fatmah H Abusharkh, Abdulrahman A Aqeel, Shaykhah S Alderaan, Norah Alabdullatif, Fahad Alshehri, Sharifa Alduraibi, Ali Alamer, Alaa Alduraibi

**Affiliations:** 1 Department of Radiology, College of Medicine, Al Qassim University, Buraydah, SAU; 2 Department of Medicine and Surgery, Al Qassim University, Buraydah, SAU; 3 Department of Medicine and Surgery, King Abdulaziz University Faculty of Medicine, Jeddah, SAU; 4 Department of Medicine and Surgery, Jazan University, Jazan, SAU; 5 Department of Medicine and Surgery, College of Medicine, Tabuk University, Tabuk, SAU; 6 Department of Radiology, Al Qassim University, Buraydah, SAU

**Keywords:** dexa scan, fragility fractures, osteoporosis, radiology, screening

## Abstract

Background

Osteoporosis is a common skeletal disorder that is characterized by low bone density and results in fragility and osteoporotic fractures; dual-energy X-ray absorptiometry (DEXA) scans play a significant role in the assessment of persons at risk of osteoporosis. Knowledge and awareness about osteoporosis and the DEXA scan is crucial in promoting early detection and prevention of osteoporosis among the general population in the Kingdom of Saudi Arabia. Our study aimed to assess the knowledge of Saudis regarding the benefits of screening for osteoporosis for early detection of abnormal bony features related to osteoporosis using the DEXA scan scoring system and prevention of osteoporotic fractures.

Methods

A cross-sectional observational study was utilized in this research, employing data from a sample of 391 participants from the general population in the Kingdom of Saudi Arabia. The participants completed self-administered online-based questionnaires with ensured anonymity. The questionnaire and the values used in this study had been previously validated.

Results

The sample for the current study primarily consisted of participants aged between 18-30 years (195, 49.9%) with a predominance of females (305, 78.0%). More than half (214, 54.7%) were non-employed, and the majority (188, 48.1%) of them were earning a monthly income of less than 5000 Riyals. Of the women participants, the majority (152, 49.8%) had more than one pregnancy, with more than half (166, 54.4%) having breastfed their children for more than six months. The study results revealed that 149 (38.1%) had a good knowledge level, while 242 (61.9%) had poor knowledge about osteoporosis screening. The results established a statistically significant association between age, gender, and occupation (p = 0.004, p = 0.002, and p = 0.001, respectively) with the level of knowledge about osteoporosis. There was no statistically significant association between region, monthly income in Riyal, number of pregnancies among women, children breastfed for more than six months and the level of knowledge about osteoporosis, with p>0.05.

Conclusion

The study revealed a considerably below-average level of knowledge about osteoporosis screening among the adult population in the Kingdom of Saudi Arabia. The knowledge about osteoporosis was found to increase with the increase in age, female participants and participants working in the medical field. The study found limited knowledge about DEXA scan and its ability to prevent osteoporotic fractures with serious concerns about its frequency of performing the procedure under normal and low bone mineral density. There is a need for collective efforts by the medical community to increase public awareness about osteoporosis and the use of DEXA scans in preventing osteoporotic fractures among the general population in Saudi Arabia.

## Introduction

Fractures and osteoporosis are major public health problems worldwide, with an increase in incidence and prevalence due to the aging of the population [1]. Osteoporosis is a common systemic skeletal disorder characterized by low bone density resulting in fragility and susceptibility to osteoporotic fractures. Osteoporosis has been called a silent disease because it is asymptomatic until a fracture complicates it; such fractures can occur at any site, but most commonly occur in the spine, hip, and wrist [2].

Osteoporosis and its complications are a major health concern in Saudi Arabia with higher mortality and morbidity among the elderly population. Dual-energy X-ray absorptiometry (DEXA) scans used for measuring bone mineral density (BMD), usually at the spine and hip, have a major role in the assessment of persons at risk of osteoporosis. Also, DEXA helps clinicians advise their patients about the applicable use of anti-fracture treatments. In comparison with other bone densitometry techniques, e.g. Quantitative Computed Tomography (QCT) and Quantitative Ultrasound (QUS), DEXA scans of the hip and spine have multiple advantages, including a consensus that BMD results can be interpreted using the World Health Organization (WHO) scores for BMD, which reflects osteoporosis, a proven way to predict patients’ fracture risk, the efficacy of anti-fracture therapy, and to monitor the response to therapy [3]. The WHO stated the values of osteoporosis, utilizing DEXA scan, as a BMD of 2.5 standard deviations (SD) or more below the mean peak bone mass, compared to the average values of young healthy adults which are calculated by the T-scores [4]. As is known, there are two common scores for BMD obtained by DEXA scan, T-scores and Z-scores. The T-score compares the results of a person's BMD with that of a healthy 30-year-old of the same sex, while the Z-score compares the results of a person's BMD with that of an average person of the same age and sex.

The current research studies point to several risk factors for osteoporosis including advanced age, history of prior fractures or parental history of osteoporosis, and low body mass index (BMI). Prominent modifiable risk factors such as tobacco consumption have shown to have a great role in developing osteoporosis. Additionally, females, especially post-menopausal women, have a higher risk of decreased bone density [5]. We conducted this study with a hypothesis that a large population of Saudi adults has a low level of knowledge.

## Materials and methods

Study design

This was a cross-sectional observational study that collected data from a population or a specific group. This study design is helpful in assessing prevalence. The primary dependent variable was participants’ knowledge score regarding osteoporosis and DEXA scans. Independent variables included age, gender, education level, and region of residence.

Study duration

This study was of a 14-month duration, starting from May 2023 until June 2024.

Study setting

The study population was in the Kingdom of Saudi Arabia, which is divided into 13 administrative regions, each divided into a number of governorates, and each governorate divided into centers. The Kingdom of Saudi Arabia is located in the southwest of Asia, with an area of 2,149,690 km^2^. The male population (Saudis and non-Saudis) is 19,240,956, and the number of females (Saudi and non-Saudi) is 14,172,704. The total population of the Kingdom during the last population census in 2018 was 33,413,660 people.

Ethical considerations

Ethical approval was obtained from the Al Qassim University Research Ethics Committee via a letter dated June 21, 2023, with approval number 607/44/17552. A consent question was added to the questionnaire; if the participant refused, the questionnaire link was closed. We ensured that the personal information of participants was preserved and confidentiality maintained.

Sample size

The sample size of this study was calculated as 385 by using the following formula for probability sampling [6].

$N=\frac{z^{2}p(1-p)}{d^{2}}$

where n is sample size, z is standard normal distribution (1.96 to a confidence level of 95%), p is anticipated population proportion (50%) for maximum sample size, and d is error not more than 0.05 (5%). Considering a 10% non-response rate, the required sample size is 425 participants.

The sampling design was a simple random sampling technique used to select participants from the general adult population in Saudi Arabia. Each individual in the target population had an equal chance of being selected.

Data management and analysis plan

The information gathered through the online survey was entered into an Excel sheet, coded, and then analyzed using Statistical Package for Social Sciences (SPSS) Version 26.0 (IBM Corp., Armonk, NY, USA). Counts and percentages were used to describe the data. Tables and figures were used to describe the information. The chi-square test was used to test associations, with a p-value of <0.05 indicating statistical significance.

Sampling technique

A convenience non-probability sampling technique was used in this study.

Inclusion criteria

Adults aged 18 years old, both male and female, who live in Saudi Arabia were selected for this study.

Exclusion criteria

People under 18 years old or with limited access to social media, those who refused to participate, and all other people who weren’t living in Saudi Arabia were excluded from this study.

Data collection methods and pilot study

An online self-administered questionnaire was constructed using Google Forms. The constructed questionnaire included many sections to achieve the objectives of the study.

A pilot study was conducted on 30 participants to test if the questionnaire’s wording is clear and understandable, as it was an Arabic translation version of the previously validated English questionnaires. The questionnaire’s reliability was calculated using Cronbach’s alpha test.

The variables used included sociodemographic factors (such as age, gender, education level, and occupation), questions regarding DEXA scan and its role in detecting low bone mineral density, and the Osteoporosis Knowledge Assessment Tool (OKAT) [7].

## Results

A total of 391 participants completed the online questionnaire, with an estimated mean age of 36.7 ± 14.5 years. The vast majority (305, 78.0%) were females, distributed across various regions in the Kingdom of Saudi Arabia. More than half (214, 54.7%) of the participants were non-employed, with almost half (188, 48.1%) earning a monthly income of less than 5000 Riyals (~1330 USD). Nearly half of the women (152, 49.8%) had experienced more than one pregnancy. Less than half (39, 45.6%) reported having breastfed their children, with 29 women (9.5%) breastfeeding once, and 110 women (36.1%) breastfeeding more than once for a duration of over six months (Table 1).

**Table 1:** Socio-demographic information of the participants (N=391) Socio-demographic information presented in frequencies (n) and proportion (%)

Sociodemographic information	Category	n (%)
Age	18-30 years	195 (49.9%)
	31-40 years	41 (10.5%)
	41-50 years	67 (17.1%)
	51-60 years	61 (15.6%)
	61-70 years	24 (6.1%)
	Above 70 years	3 (0.8%)
Gender	Female	305 (78.0%)
	Male	86 (22.0%)
Region	Central region	76 (19.4%)
	Eastern region	85 (21.7%)
	Northern region	73 (18.7%)
	Southern region	71 (18.2%)
	Western region	18 (22.0%)
Occupation	Employed in the medical field	71 (18.2%)
	Employed outside the medical field	106 (27.1%)
	Non-employed	214 (54.7%)
Monthly income in Saudi Riyals	<5000	188 (48.1%)
	>10000	82 (21.0%)
	5000-10000	121 (30.9%)
Number of pregnancies (for women)	More than once	152 (49.8%)
	Once	22 (7.2%)
	None	131 (43.0%)
Children breastfed for more than 6 months (for women)	More than once	110 (36.1%)
	Once	29 (9.5%)
	None	166 (54.4%)

Table 2 demonstrates the knowledge about the DEXA scan and its ability to detect osteoporosis for the prevention of osteoporotic fractures. Only 114 (29.2%) of the participants had heard about the DEXA scan. Out of 114 respondents, the vast majority (105, 92.1%) were aware that DEXA scan is used for scanning bone mineral density, with a substantial proportion (53, 46.5%) correctly noting that no precautions are required for the procedure. More than half of the participants correctly answered the type of procedure used in a DEXA scan as an X-ray (58 participants, 50.9%), and also correctly recognized the recommended ages for undergoing the scan - 65 years for women and 70 years for men (61 participants, 53.5%). Additionally, a considerable proportion (47, 41.2%) of the respondents were aware of the role of DEXA scans in identifying low bone density, which supports early intervention to prevent osteoporotic fractures. However, more than half of the respondents were not sure of the frequency of performing DEXA scans under normal and low bone mineral density.

**Table 2:** Knowledge regarding DEXA scan Knowledge regarding DEXA scan presented in frequencies (n) and proportion (%)

Questions	Categories	n (%)
Have you heard about DEXA scan?	Yes	114 (29.2%)
	No	277 (70.8%)
What is DEXA scan used for?	A scan for bone mineral density	105 (92.1%)
	A scan for tumors	6 (12.3%)
	I don’t know	3 (2.6%)
Are there any safety measures/ precautions needed for DEXA scan?	Yes	61 (53.5%)
	No	53 (46.5%)
What is the procedure type?	MRI	8 (7.0%)
	Nuclear imaging	20 (17.5%)
	X-ray	58 (50.9%)
	I don’t know	28 (24.6%)
What is the recommended age for DEXA scan?	Healthy women at 25 years old, and men at 30 years old	5 (4.4%)
	Healthy women at 35 years old and men at 40 years old	21 (18.4%)
	Women at 65 years old, men at 70 years old	61 (53.5%)
	I don’t know	27 (23.7%)
How often can the scan be performed for normal BMD?	Annually	8 (7.0%)
	Every 15 years	5 (4.4%)
	Every 5 years	44 (38.6%)
	I don’t know	57 (50.0%)
How often should the scan be performed for low BMD?	Annually	35 (30.7%)
	Every 2 years	13 (11.4%)
	Every 3 years	18 (15.8%)
	I don’t know	48 (42.1%)
Can DEXA scan help prevent osteoporotic fractures?	Yes	47 (41.2%)
	No	30 (26.3%)
	I don’t know	37 (32.5%)

Table 3 depicts the knowledge about osteoporosis among participants, using the Osteoporosis Knowledge Assessment Tool (OKAT) [7]. More than half of the participants were aware that osteoporosis leads to an increased risk of bone fractures (351, 89.8%), cigarette smoking can contribute to osteoporosis (222, 56.8%) and that a fall is just as important as low bone density in causing fractures (240, 61.4%). The majority (278, 71.1%) of the participants knew that many women develop osteoporosis at 80 years old with a substantial majority (169, 43.2%) correctly noting that by the age of 50 women can expect at least one bone fracture before they die. More than half of the participants were aware that it is easy to predict whether a person is at risk of osteoporosis by their clinical risk factors (205, 52.4%), family history of osteoporosis strongly predisposes one to osteoporosis (240, 61.4%), an adequate calcium intake can be achieved from two glasses of milk a day (234, 59.8%), and that sardines and broccoli are good sources of calcium for people who cannot take dairy products (266, 68.0%). However, the majority of the participants were not sure whether white women are at the highest risk of fracture as compared to other races (304, 77.7%), that the consumption of alcohol in moderation has little effect on osteoporosis (291, 74.4%) and that high salt intake is a risk factor for osteoporosis (227, 58.1%).

**Table 3:** Knowledge regarding osteoporosis Knowledge regarding osteoporosis presented in frequencies (n) and proportion (%)

Questions	Categories	n (%)
Osteoporosis can increase the risk of bone fractures	True	351 (89.8%)
	False	6 (1.5%)
	Don’t know	34 (8.7%)
Osteoporosis usually causes symptoms (e.g., pain) before bone fractures	True	291 (74.4%)
	False	35 (9.0%)
	Don’t know	65 (16.6%)
Having a higher bone mass at the end of childhood age provides no protection against the development of osteoporosis later in life	True	232 (59.3%)
	False	23 (5.9%)
	Don’t know	136 (34.8%)
Osteoporosis is more common in males	True	38 (9.7%)
	False	241 (61.6%)
	Don’t know	61 (16.8%)
Smoking can contribute to the development of osteoporosis	True	222 (56.8%)
	False	39 (10.0%)
	Don’t know	130 (33.2%)
White women have the highest risk of fractures as compared to other races	True	87 (22.3%)
	False	89 (22.8%)
	Don’t know	215 (55.0%)
A fall is just as equally important as low bone strength in provoking fractures	True	240 (61.4%)
	False	54 (13.8%)
	Don’t know	97 (24.8%)
By the age of 80, the majority of women will have osteoporosis	True	278 (71.1%)
	False	29 (7.4%)
	Don’t know	84 (21.5%)
By the age of 50, most women may expect at least one fracture before they die	True	169 (43.2%)
	False	68 (17.4%)
	Don’t know	154 (39.4%)
Any type of physical activity is beneficial against osteoporosis	True	167 (42.7%)
	False	77 (19.7%)
	Don’t know	147 (37.6%)
It is easy to predict whether I am at risk of developing osteoporosis by my clinical risk factors	True	205 (52.4%)
	False	57 (14.6%)
	Don’t know	129 (33.0%)
Family history of osteoporosis strongly predisposes to developing osteoporosis	True	240 (61.4%)
	False	41 (10.5%)
	Don’t know	110 (28.1%)
An adequate calcium intake can be obtained from drinking two glasses of milk a day	True	234 (59.8%)
	False	57 (14.6%)
	Don’t know	100 (25.6%)
Broccoli and sardine are great sources of calcium for people who are intolerant to dairy products	True	266 (68.0%)
	False	24 (6.1%)
	Don’t know	101 (25.8%)
Taking calcium supplements alone can prevent bone loss	True	105 (26.9%)
	False	186 (47.6%)
	Don’t know	100 (25.6%)
Moderate alcohol intake has little effect on osteoporosis	True	100 (25.6%)
	False	132 (33.8%)
	Don’t know	159 (40.7%)
A high sodium intake is considered a risk factor for osteoporosis	True	164 (41.9%)
	False	38 (9.7%)
	Don’t know	189 (48.3%)
There is a little amount of bone loss in the 10 years following menopause	True	195 (49.9%)
	False	46 (11.8%)
	Don’t know	150 (38.4%)
Hormone replacement therapy prevents further bone loss at any age after the onset of menopause	True	135 (34.5%)
	False	49 (12.5%)
	Don’t know	207 (52.9%)
There are no effective treatments available for osteoporosis in Saudi Arabia	True	88 (22.5%)
	False	143 (36.6%)
	Don’t know	160 (40.9%)

Table 4 presents the relationship between participants’ socio-demographic information and their level of knowledge about osteoporosis.

**Table 4:** The association between socio-demographic information and level of knowledge regarding osteoporosis * Significant at p<0.05 level

	Level of knowledge			
Variables	Category	Poor	Good	p-value
Age	18-30 years	131 (67.2%)	64 (32.8%)	0.004*
	31-40 years	27 (65.9%)	14 (34.1%)	
	41-50 years	43 (64.2%)	24 (35.8%)	
	51-60 years	36 (59.0%)	25 (41.0%)	
	61-70 years	14 (58.3%)	10 (41.7%)	
	Above 70 years	2 (66.7%)	1 (33.3%)	
Gender	Female	125 (41.0%)	180 (59.0%)	0.002*
	Male	45 (52.3%)	41 (47.7%)	
Region	Central region	49 (64.4%)	27 (35.6%)	0.156
	Eastern region	52 (61.2%)	33 (38.8%)	
	Northern region	49 (67.1%)	24 (32.9%)	
	Southern region	48 (67.6%)	23 (32.4%)	
	Western region	12 (66.7%)	6 (33.3%)	
Occupation	Employed in the medical field	31 (43.7%)	40 (56.3%)	0.001*
	Employed outside the medical field	63 (59.4%)	43 (40.6%)	
	Non-employed	138 (64.5%)	76 (35.5%)	
Monthly income in Saudi Riyals	<5000 (~1330 USD)	120 (63.8%)	68 (36.2%)	0.253
	>10000 (~2660 USD)	55 (67.1%)	27 (32.9%)	
	5000-10000 (~1330-2660 USD)	75 (62.0%)	46 (38.0%)	
Number of pregnancies (for women)	More than once	93 (61.2%)	59 (38.8%)	0.269
	None	89 (67.9%)	42 (32.1%)	
	Once	14 (63.6%)	8 (36.4%)	
Children breastfed for more than 6 months (for women)	More than once	68 (61.8%)	42 (38.2%)	0.114
	None	114 (68.7%)	52 (31.3%)	
	Once	18 (62.1%)	11 (37.9%)	

The results established a statistically significant association between age, gender, and occupation (p = 0.004, p = 0.002, and p = 0.001, respectively) with the level of knowledge about osteoporosis.

Logistic regression analysis revealed that non-employed participants had significantly lower odds of having good knowledge about osteoporosis compared to those employed in the medical field (OR = 0.49, 95% CI: 0.27-0.91, p = 0.024). Gender and age were not statistically significant predictors, although male participants tended to have lower odds of good knowledge (OR = 0.58, p = 0.071).

There was no statistically significant association between region, monthly income in Riyal, number of pregnancies among women, children breastfed for more than 6 months, and the level of knowledge about osteoporosis (p>0.05).

The study results revealed a good knowledge level about osteoporosis in 149 (38.1%) of the participants, while a poor knowledge level was found in 242 (61.9%) of the participants.

## Discussion

The study aimed to assess the level of knowledge of Saudi adults regarding osteoporosis and the role of DEXA scan in the prevention of osteoporotic fractures. The sample for the current study primarily consisted of participants aged between 18-30 years, with a predominance of females; the majority of them were single, non-employed, and earning a monthly income of less than 5000 Riyals.

The findings revealed that only 149 (38.1%) of the participants had good knowledge about osteoporosis. This shows the existence of insufficient knowledge about osteoporosis among the population in Saudi Arabia which is similar to the findings of a study conducted by Beshyah et al. in the United Arab Emirates which established poor knowledge regarding osteoporosis among practicing physicians [8]. Similarly, one study conducted by Alshareef et al. among young college women at the University of Riyadh in Saudi Arabia found a good osteoporosis knowledge score in only 8% of the participants [9]. The study conducted by Sitati et al. found limited osteoporosis knowledge in 45.2% of the participants in Kiambu District in Kenya [10]. The difference in knowledge level about osteoporosis observed in the study compared to other previous studies is likely attributed to the small sample and the heterogeneity of the current study population. The study by Barzanji et al. reported poor knowledge levels and gaps in knowledge of osteoporosis and its application; this highlights the need to improve preventive health education to promote health practices and behaviors [11].

According to the study findings, there was a considerable level of knowledge demonstrated by more than half of the participants regarding the risk of bone fractures caused by osteoporosis (351, 89.8%) and the contribution of cigarette smoking to osteoporosis (222, 56.8%) as well as the effects of a fall and low bone strength on bones and fractures (240, 61.4%). Additionally, more than half of the participants exhibited awareness that clinical risk factors can help to identify a person who is at risk of osteoporosis (205, 52.4%), and the family history of osteoporosis strongly predisposes one to osteoporosis (240, 61.4%). The findings concur with those of the study conducted by Alghadir et al. which found family history to be a significant risk factor for osteoporosis [12].

A statistically significant association was established between age, gender, occupation, and the level of knowledge about osteoporosis. The study revealed a growth in good knowledge with an increase in the age of the participants. It was revealed that female participants showed a higher level of knowledge about osteoporosis than the male participants. The findings of the study are consistent with those of the study conducted by Ensrud et al. which established the link between osteoporosis knowledge and increasing age, female gender, and ethnicity [13]. Similarly, the study conducted by Ramírez et al. established an association between osteoporosis knowledge and female gender and race with White ethnic background being more at risk of osteoporosis than Blacks [14]. Additionally, participants who were employed in the medical field showed significantly higher levels of knowledge about osteoporosis than those employed in other fields and the non-employed. The findings of the study mirror the findings of the study conducted by Saeedi et al. which revealed a high level of osteoporosis knowledge (between 36.5% and 92.2%) among physicians [15].

Regarding the knowledge about the DEXA scan and its abilities to detect osteoporosis for the prevention of osteoporotic fractures, only 114 (29.2%) of the participants had heard about the DEXA scan with a considerable proportion of them being aware of its procedure type and the recommended age for performing the scan procedure. Furthermore, the majority of the respondents who had heard about DEXA scans were not sure of the frequency of performing the procedure under normal and low bone mineral density. This shows limited knowledge regarding the DEXA scan with a substantial proportion of the participants not aware of the scan's ability to prevent osteoporotic fractures. The study noted gaps in knowledge that need to be addressed in adopting a public health education program to increase knowledge and awareness about osteoporosis among the general public in the Kingdom of Saudi Arabia. To our knowledge, this is one of the few studies in Saudi Arabia to study the public's understanding of DEXA scans in the context of osteoporosis prevention.

There are some limitations to the results of this study. A cross-sectional observational study has a limitation attributed to its inability to assess causal relationships. Additionally, considering that the study involved the administration of online questionnaires, the study relied on respondents accurately documenting their responses without the ability to check this, which may have contributed to a potential bias.

A hospital-based study estimated the prevalence of osteoporosis in the Saudi population as 63.6% in men and 58.4% in post-menopausal women [16]. Additionally, fragility fractures are resulting in serious consequences including risk of early death and limitation in physical functioning, ultimately resulting in a decline in quality of life [17]. In a study conducted by Saleh et al., the incidence of hip fracture among Saudi adults >50 years old was 2,949 in the year 2015. It is predicted to increase to approximately 20,328 in 2050 [18]. In another study conducted in 2020, among 400 postmenopausal women who were referred for BMD assessment using DEXA, the prevalence of vertebral fracture was 65.2%, and among those subjects with vertebral fracture, 59% had osteoporosis at either their spine or femoral neck [19]. In 2019, Saudi Arabia predicted to incur SR2.38 billion ($636 million; $1.55 billion PPP) for 174,225 osteoporotic fractures; thus, reducing the fracture burden is essential [20].

Given the low level of public awareness regarding osteoporosis and the role of DEXA scans in its early detection and prevention, future studies should aim to explore targeted educational interventions and their effectiveness in improving knowledge and attitudes.

## Conclusions

The study revealed a considerably below-average level of knowledge about osteoporosis among the adult population in the Kingdom of Saudi Arabia. The knowledge about osteoporosis was found to increase with an increase in age, female participants, and participants working in the medical field. The study found limited knowledge about DEXA scan and its ability to prevent osteoporotic fractures with serious concerns about its frequency of performing the procedure under normal and low bone mineral density. There is a need for collective efforts by the medical community to increase public awareness about osteoporosis and the use of DEXA scans in preventing osteoporotic fractures among the general population in Saudi Arabia.
